# Perinatal Health Inequalities in the Industrial Region of Estonia: A Birth Registry-Based Study

**DOI:** 10.3390/ijerph191811559

**Published:** 2022-09-14

**Authors:** Usha Dahal, Triin Veber, Daniel Oudin Åström, Tanel Tamm, Leena Albreht, Erik Teinemaa, Kati Orru, Hans Orru

**Affiliations:** 1 Institute of Family Medicine and Public Health, University of Tartu, 50411 Tartu, Estonia; 2 Institute of Social Science, University of Tartu, 51003 Tartu, Estonia; 3 Section of Sustainable Health, Umeå University, 901 87 Umea, Sweden; 4 Environmental Health Department, Estonian Health Board, 10617 Tallinn, Estonia; 5 Estonian Environmental Research Centre, 10617 Tallinn, Estonia

**Keywords:** inequality, air pollution, socioeconomic status, industrially contaminated sites, adverse birth outcomes, preterm birth, low birth weight

## Abstract

Despite the increasing number of studies on industrially contaminated sites (ICS) and their health effects, there are very few studies on perinatal health outcomes in ICSs. In the present study, we examined the perinatal health inequalities by comparing adverse birth outcomes (ABOs) in the oil shale industry region of Ida-Viru County in Estonia with national-level figures and investigated the effects of maternal environmental and sociodemographic factors. Based on the 208,313 birth records from 2004–2018, Ida-Viru ICS has a birth weight 124.5 g lower than the average of 3544 g in Estonia. A higher prevalence of preterm birth (4.3%) and low birth weight (4.8%) in Ida-Viru ICS is found compared to 3.3% on both indicators at the national level. Multiple logistic regression analysis shows the statistically significant association of ABOs with fine particle (PM_2.5_) air pollution, mother’s ethnicity, and education throughout Estonia. However, in Ida-Viru ICS, the ABOs odds are remarkably higher in these characteristics except for the mother’s ethnicity. Furthermore, the ABOs are associated with the residential proximity to ICS. Thus, the Ida-Viru ICS has unequally higher odds of adverse perinatal health across the environmental and sociodemographic factors. In addition to reducing the air pollutants, policy actions on social disparities are vital to address the country’s unjustly higher perinatal health inequalities, especially in the Ida-Viru ICS.

## 1. Introduction

Health is affected by numerous individual, social, and environmental characteristics [1,2]. The socio–environmental settings have an important role in shaping the individual lifestyle and behavior, including the biological consequences, which in turn influence health outcomes and health inequality [1,2,3]. Air pollution from industrially contaminated sites (ICS) is identified as one of the major environmental exposures affecting health, resulting in numerous adverse health outcomes, including reduced newborns’ health in terms of adverse birth outcomes (ABOs) [4]. Furthermore, ICS is often the hub of multiple environmental stressors, while pregnancy and newborn stages are identified as the most susceptible life stages with high risks for health effects [5,6,7]. ABOs are considered a serious public health problem as they increase the probability of short- and long-term health consequences, including chronic diseases in later life stages [8,9,10,11,12,13].

Systematic reviews have shown significant socioeconomic disparities in ABOs [14,15]. ABOs are also used as one of the health indicators to measure existing health inequalities [16,17]. Studies suggest several factors that affect unequal birth outcomes, varying not only from individual pregnant maternal health status, but also from prenatal exposure to air pollutants [18,19], residential proximities to ICS [20,21,22,23,24], and lower maternal and neighborhood socioeconomic status (SES) [14,25].

Studies focusing specifically on ICS and ABOs are few; however, a recent systematized review on industrial air pollutants found that birth outcomes are affected by maternal exposure to industrial air pollutants and living closer to industrial areas [26]. Considerable evidence on fine particles (PM_2.5_) often emitted in higher concentrations in ICS, has shown associations with low birth weight (LBW) [18,21,22,27,28] and preterm birth (PTB) [21,22,27,29]. Liu and colleagues’ [30] meta-analysis showed that even a low level of PM_2.5_ is associated with PTB. Similarly, numerous studies on particulate matter (PM_10_) from industrial sources have shown the effect on LBW [18,31,32,33,34] and PTB [18,27,29,34,35].

A growing body of literature on spatial indicators suggests a strong association between proximity to power plants and LBW [21,22,24,36,37], and PTB [20,21,22,23,27]. Higher risks of LBW and PTB are reported in the proximity to coal-fired power plants [22], oil shale gas drilling [38], oil refineries [39,40], petrochemical facilities [28,32,41,42,43], coke and steel industries [44]. Moreover, industrial areas hosting several facilities have also shown increasing LBW [45].

Often, the socioeconomically deprived or marginalized population tends to live in or close to the industrially contaminated sites [46,47]. Studies have shown that both neighborhood- and individual-level socioeconomic status are associated with ABOs [14,25,48,49,50,51]. A mother’s education and ethnicity/race are associated with PTB and LBW [50]. Across socioeconomically different areas, PTB is found to associate with residents with low education, high unemployment, and high poverty [51]. Messer et al. [51] found that racial segregation affected the PTB in the United States, whereas in Canada, Auger et al. [48] found that the social class indicators: maternal birthplace and education, affected birth outcomes instead of race. Nevertheless, mostly perinatal health inequalities in high–income countries are persistent among women with lower SES, especially with lower education and in regions that are socially and physically deprived [49].

In terms of the individual factors, maternal health status also affects birth outcomes, for instance, chronic conditions such as diabetes, hypertension, increased blood sugar during pregnancy, and preeclampsia [52,53]. Advanced maternal age has shown poor birth outcomes and maternal complications exacerbating the ABOs [54,55]. In addition, air pollutants can also worsen ABOs among the mothers with complications [53].

Despite the growing literature on the health impacts of ICS [56], there are only a few studies on health inequality [4], particularly those focusing on the perinatal period, which is one of the most vulnerable life stages [5,6,7].

In this study, we focus on the perinatal health inequalities in an ICS, the oil shale industry region of Ida-Viru County in Estonia. Estonia is one of the highest greenhouse gas emitters per capita in OECD [57,58,59] and has one of the world’s highest ecological carbon footprints per capita [60] due to the oil shale industries located in Ida-Viru County. Despite the decreasing emission over the past years, pollutant concentrations have occasionally been exceeding the limit values [57,59]. Residents in Ida-Viru also more often report health effects varying from respiratory and heart–related syndromes, including annoyance, compared to residents in non-oil shale areas [61]. In addition, asthma prevalence among children [62] and lung cancer incidence is higher in oil shale areas in Ida-Viru County [63]. Nevertheless, Kanger and Sovacool [64] have concluded that environmental health effects are not well–recognized, potentially leading to worsening health injustice.

In addition to the inequalities in environmental exposures, Ida-Viru also has lower SES, and specific ethnicities (non–Estonians, mainly Russians) dominate the county [65]. Generally, Russian minorities have poor SES and a weak sense of belonging to their respective living countries [66]. Given such socioeconomic context and industry location, the Ida-Viru population becomes a disadvantaged group with a disproportionate exposure level of health-harming characteristics [67,68].

In this article, we aim to explore the perinatal health inequalities across the different populations in the Ida-Viru ICS. We adopted Dahlgren and Whitehead’s definition of health inequalities as the socially produced systematic and unjust differences in health across the population [1]. We will focus on the following specific research questions:

How does maternal exposure to air pollutants affect birth outcomes in Estonia? How does Ida-Viru ICS differ from the nationwide Estonian figure?

How does the maternal residential proximity to ICS affect birth outcomes?

How are the maternal sociodemographic characteristics associated with ABOs? Moreover, how different is Ida-Viru ICS in these characteristics?

## 2. Materials and Methods

### 2.1. Study Population and Variables

In the current study, birth registry data on singleton births were obtained from the Estonian Medical Birth Registry from 1 January 2004 to 31 December 2018. All registered birth in Estonia include data on maternal and neonatal characteristics, including demographics. Neonatal characteristics obtained from the birth registry included personal identification code, date of birth, gender, live or stillbirth, gestation days, and birth weight. In Estonia, gestational age is predominantly determined by ultrasound, and birth weight is measured immediately after the baby is born. The ABO indicators used in the article are preterm birth (PTB: ≤37 weeks of gestation) and low birth weight (LBW; birth weight ≤ 2500 g). LBW and PTB were dichotomously coded and used as the birth outcome indicators. Maternal sociodemographic characteristics and maternal health status that are identified or suspected confounders for ABOs obtained from the birth registry are the level of education, ethnicity, age, preeclampsia, in vitro fertilization, earlier cesarean delivery, preterm birth risk during pregnancy, miscarriage risk during pregnancy, hypertension, chronic diabetes, and gestational diabetes. Data on smoking during pregnancy recorded in the birth registry were excluded due to the self–reported data reliability issue. The county-level income coefficient from 2004–2018 from Statistics Estonia was obtained and used as an indicator of neighborhood SES.

### 2.2. Air Pollution Exposure Assessment

**Figure 1:**
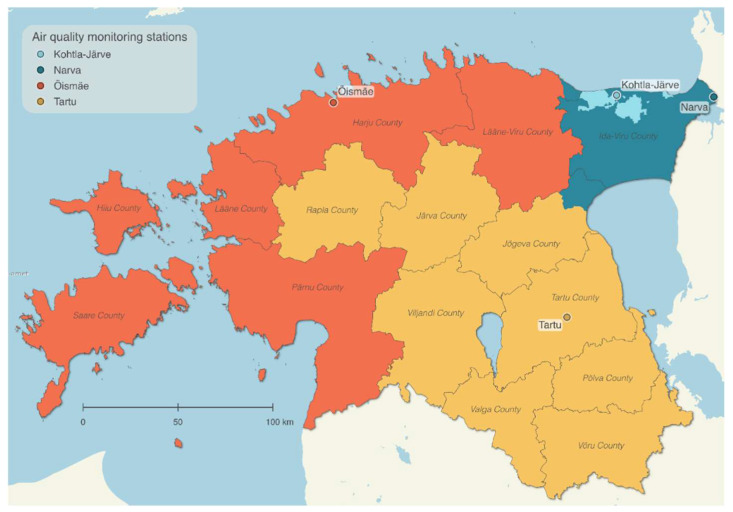
Applied air quality monitoring stations and their represented areas for subsequent temporal trend. Basemap: © Land Board.

The exposure assessment included two levels of assessment: a combined method using measured and modeled data to catch temporal and spatial differences, and residential proximity to the oil shale industry. For the combined method of measuring and modeling the air pollution, first, the measured particulate matter (PM_10_), fine particles (PM_2.5_), and nitrogen dioxide (NO_2_) concentrations were calculated for each child according to daily average data in the nearest representative urban background air quality monitoring station (Figure 1) during the first and last trimester (gestational weeks 1–13 and from 28 until birth, respectively). Second, the annual mean concentrations of PM_10_, PM_2.5_, and NO_2_ were modeled in 1 × 1 km grids using an Eulerian air quality dispersion model that formed part of the Airviro Air Quality Management System (Apertum IT AB, v5.01, http://airviro.com (accessed on 5 January 2022)); see the detailed description in the Airviro Documentation [69]. Industrial pollutant emission levels in 2017 were retrieved from the ambient air emission database OSIS2017, which consists of annual emissions (tons per year) of more than 40 different pollutants and substance classes (incl. PM_10_, PM_2.5_ and NO_2_) reported by companies and validated by the Estonian Environmental Board. Same year transport–sector emissions came from the traffic emissions database Traffic2017, domestic heating emissions from the local heating emissions database Localheating2017, both compiled by Estonian Environmental Research Center, and agriculture emissions. The modeled concentrations were validated with measurement data obtained from the monitoring stations all over Estonia and with concentrations measured by passive samplers. For visualization, the model output was generalized and classified into seven classes, using the ArcGIS (v10.3, ESRI, Redlands, CA, USA) scripting environment ArcPy. In ArcGIS, the annual concentrations of modeled pollutants per grid cell were linked with the geocoding of each child’s home address at the moment of birth. During the geocoding, the geographical coordinates of the address points were found according to the textual addresses. The public geocoding service of the Land Board was applied, which is available on the website as Address Data Geocoding [70]. Third, the measured concentrations were adjusted according to the difference in modeled concentration between levels at the home address and in the nearest temporal trend in the representative monitoring station. For measuring and modeling the pollutants, the following monitoring stations were used as shown in Figure 1: Tallinn Õismäe (Baltic Sea bordering Counties), Tartu (inland Counties), and Narva and Kohtla–Järve (municipalities in Ida-Viru County).

**Figure 2:**
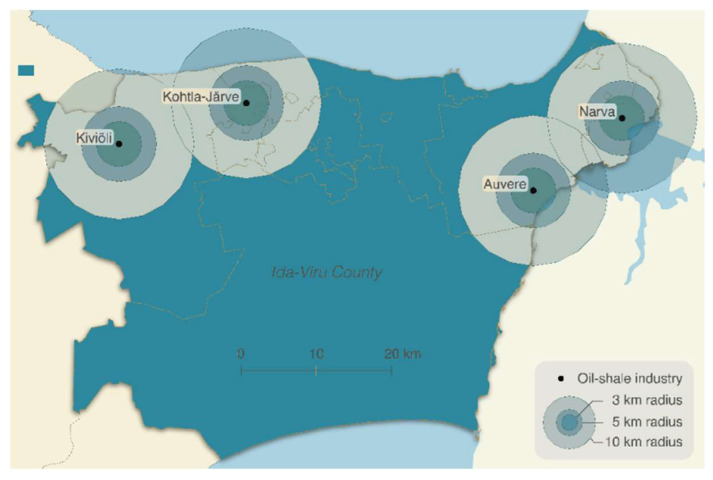
Oil shale industries in Ida-Viru County and 3 km, 5 km, and 10 km buffers around them. Basemap: © Land Board.

As the alternative exposure assessment variable, proximity to the nearest oil shale industry from home was calculated for each newborn. According to the proximity, children were divided into the following groups: ≤3 km, ≤5 km, ≤10 and ≥10 km (Figure 2). Among 208,313 newborns, 1112 were excluded while measuring the effect of air pollution and proximity because of data unavailability on the address.

### 2.3. Statistical Analysis

First, we compared the prevalence of ABOs and their distribution in relation to social markers of Ida-Viru County (newborns whose mother’s place of residence was indicated in the birth register as Ida-Viru County) with the national level. Second, multiple logistic regression analysis was performed to analyze the relationship between birth outcomes and exposure to air pollutants (PM_10_, PM_2.5_, and NO_2_), proximities to oil shale industrial sites (≤3 km, ≤5 km, and ≤10), and maternal sociodemographic characteristics (ethnicity, education, and age). Logistic regression comprised three models: first, the crude model for the association between exposure and outcome variables; a second adjusted model for the individual-level sociodemographic variables (mother’s education, ethnicity, and age), a third fully-adjusted model adjusting for the individual–level sociodemographic variables including the status of pregnant mother (mother’s education, ethnicity, age, in vitro fertilization, earlier cesarean section, preeclampsia, preterm birth risk during pregnancy, miscarriage risk during pregnancy, gestational diabetes, mother’s hypertension, and chronic diabetes). The third model is also adjusted for neighborhood SES in the analysis with residential proximity and social markers.

## 3. Results

### 3.1. Distribution of Adverse Birth Outcomes of Singleton Births in Ida-Viru County and Estonia

A total of 208,313 births were registered from 2004–2018, of which 18,626 births were recorded in Ida-Viru County. In contrast to the national figure of 25%, almost 80% of mothers in Ida-Viru County identified themselves as having a Russian ethnic background (Table 1). In Ida-Viru County, the greatest share of mothers had an applied education (42.3%), and a smaller share had a higher education (18.1%) compared to the national level of 30.6% and 31.1%, respectively. The mothers were younger, when giving birth, in Ida-Viru County compared to the Estonian average. The decreasing delivery trend with increasing age can be observed all over Estonia; however, the trend was lighter in Ida-Viru County. Among 11,033 women residing within ≤10 km proximity to oil shale industries, 99.6% were from Ida-Viru County as the county hosts all of the oil shale industries of Estonia.

Table 1 illustrates that compared to national figures, Ida-Viru County had a higher prevalence of ABOs in all sociodemographic categories, including mother’s education and age. At the national level, Russians (4% LBW, 3.6% PTB) and other non-Estonian mothers (4.1% LBW, 3.6% PTB) shared higher percentages of ABOs than Estonians (3% LBW, 3.1% PTB). However, in Ida-Viru County, the share of ABOs was slightly higher among only Russians (5% LBW, 4.3% PTB) compared to Estonians. In terms of residential proximity to oil shale industries, the distribution of ABOs was increasing with decreasing proximity.

**Figure 3:**
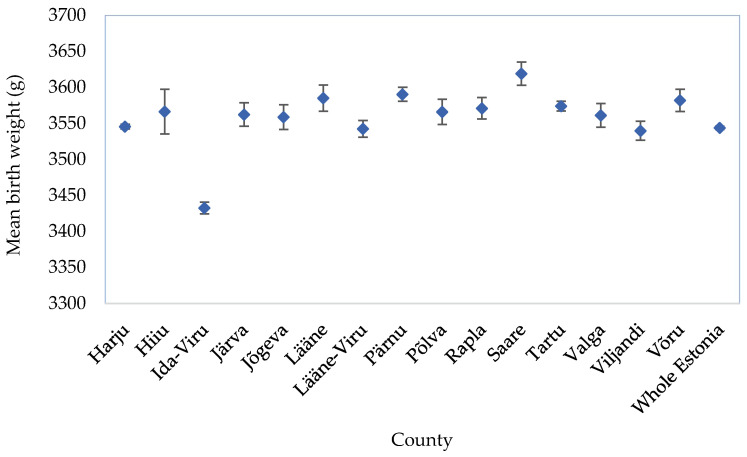
National and county–level average birth weight (grams) with a 95% confidence interval.

At the national level, the prevalence of LBW and PTB was 3.3% for both indicators; however, Ida-Viru County has a higher prevalence: 4.8% for LBW and 4.3% for PTB (Table 1). Furthermore, Figure 3 depicts that the average birth weight was lower in Ida-Viru County than in any other county and 124.5 g lower than the national average of 3544 g.

### 3.2. Air Pollution Exposure during Pregnancy

**Figure 4:**
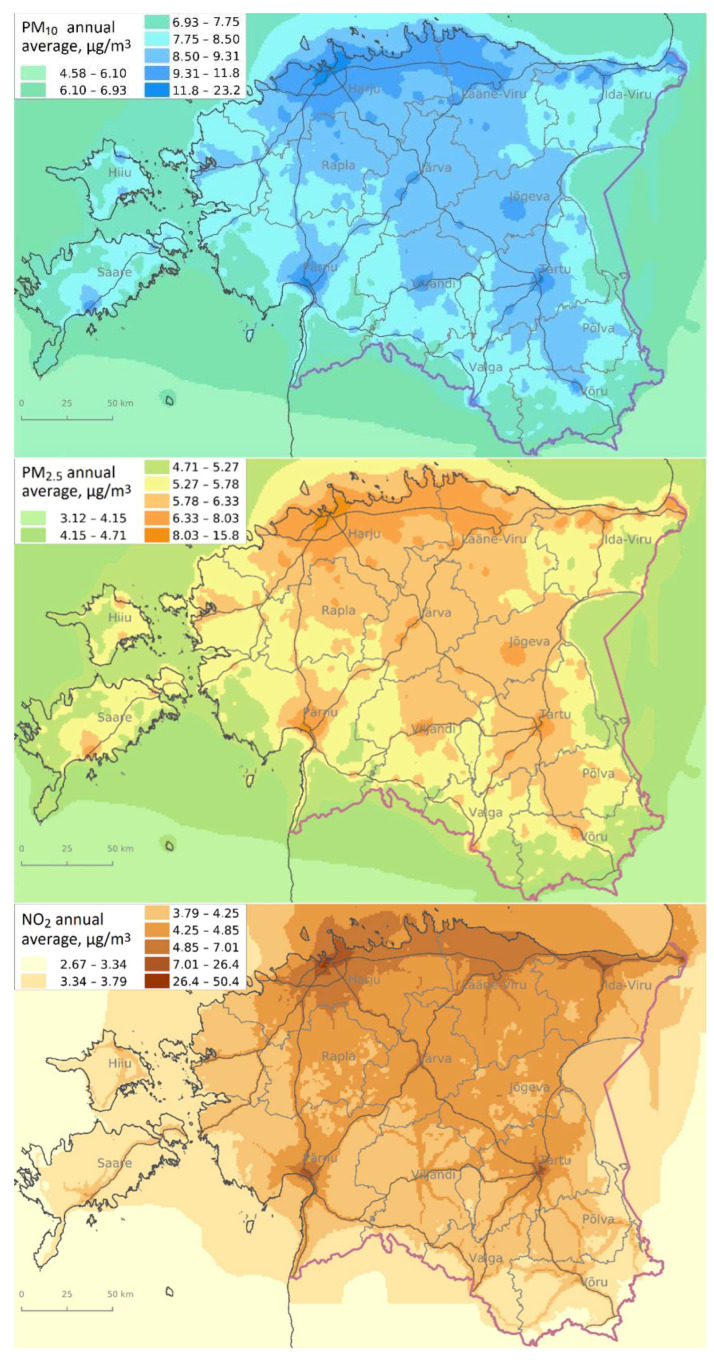
Modeled annual average concentrations of particulate matter (PM_10_), fine particles (PM_2.5_), and nitrogen dioxide (NO_2_) in Estonia. Basemap: © Maaamet.

The modeled annual average concentrations of air pollution (Figure 4) show a higher level of pollutants, primarily in larger cities, Northern Estonia, and the industrial areas of Ida-Viru County. Higher PM_2.5_ concentrations were widely dispersed in Northern Estonia, and the highest NO_2_ concentrations were found in the center of Tallinn.

The annual average concentrations of PM_10_, PM_2.5_, and NO_2_ in four air quality monitoring stations show a decreasing trend in 2004–2017; however, the concentrations significantly increased again in 2018. The concentration in Tallinn–Õismäe, Tartu, Kohtla–Järve, and Narva monitoring stations are presented in Supplementary Material Figure S1. Two stations, Kohtla–Järve and Narva, are located in Ida-Viru County. The level of PM_2.5_ was mostly higher than the recommended World Health Organization (WHO) air quality guideline level (5 µg/m^3^) [71], except for fluctuating trends between 2015–2017. Apart from the Tartu station, PM_10_ remained below the recommended level (15 µg/m^3^) [71] since 2015; however, the trend mostly fluctuated on the borderline. NO_2_ was below the WHO recommended level of 10 µg/m^3^ [71] in Kohtla–Järva throughout the period; nevertheless, the level in Narva was mostly borderline, but the peak was in 2010, with a sharp decline in 2017.

In general, a relatively similar level of prenatal exposure to average concentration of PM_10_, PM_2.5_, and NO_2_ can be observed among the newborns in different groups (term birth with normal weight, LBW, and PTB) over the period 2004–2018 during the first and third trimesters (Table S1). However, compared to the national level, exposure to PM_2.5_ and PM_10_ was slightly higher, and NO_2_ was slightly lower in Ida-Viru County. The discrepancy observed between the modeled air pollution concentrations (Figure 4) and the average annual exposure to air pollutants (Table S1) is due to the population density: urban population in Ida-Viru is larger, whereas the population is more dispersed in rural areas in the rest of the Estonian counties.

### 3.3. Impact of Air Pollution, Residential Proximity, and Sociodemographic Factors on Adverse Birth Outcomes

This section presents the results on how air pollutants, residential proximity, and sociodemographic factors are affecting ABOs.

Table 2 shows that exposure to PM_2.5_ during the first trimester increased the chance of PTB by 12% (OR = 1.12, 95% CI: 1.02–1.23) per 10 µg/m^3^ increase in exposure; however, in the crude model (Table S2), the association was insignificant. A similar increase in PM_2.5_ exposure during the third trimester showed a significant association with LBW in Ida-Viru County (OR = 1.56, 95% CI: 1.16–2.08), whereas the association was insignificant at the national level. Exposure to PM_10_ was not significantly associated with ABOs at both the national and county levels in all models. In the crude model, significant opposite association with LBW with NO_2_ was shown (Table S2) but not in the adjusted and fully–adjusted models. Further analysis of other chemicals benzo(a)pyrene (Table S3) exposure during whole pregnancy, showed a significant association with only LBW (OR = 4.06, 95% CI: 1.50–11.1 per 10 ng/m^3^ increase in exposure).

The crude and adjusted model (Table S4 and Table 3) showed the significant association of ABOs with all the distances (≤3, ≤5, ≤10) we tested for compared to >10 km. However, in a fully adjusted model for PTB, a significant association was found with the proximity of ≤3 km (OR = 1.58, 95% CI: 1.26–1.98), and the association did not change much from the adjusted model. In contrast, LBW was significantly associated with ≤3 km (OR = 1.51, 95% CI: 1.21–1.88) and ≤5 km (OR = 1.18, 95% CI: 1.04–1.33). For ≤10 km, the association remained significant, when adjusted with the mother’s sociodemographic variables (OR = 1.42, 95% CI: 1.29–1.57), but when adjusted with both mother’s sociodemographic variables and maternal health status, the association became insignificant. The association decreased with increasing distance from the oil shale industries, although not significant with ≤5 and ≤10 km for PTB and ≤10 km for LBW in fully adjusted models.

Table 4 shows that the Russian mothers had higher odds of LBW at both national and Ida-Viru County levels, while mothers from other non–Estonian ethnicities also had higher odds of LBW at the national level. Significant associations between PTB and ethnicity were shown only in the crude model (Table S5) and adjusted model with the mother’s age and education (OR = 1.15, 95% CI: 1.09–1.21) at the national level, but the associations with LBW were shown in the fully adjusted model (OR = 1.29, 95% CI: 1.22–1.36) as well. A different pattern was seen in Ida-Viru County, where a significant association was not shown with PTB but only with LBW among Russian mothers (OR = 1.29, 95% CI: 1.05–1.57). However, at both the national and Ida-Viru County level, Russian mothers had a 29% higher chance of having LBW babies than Estonian mothers. Furthermore, mothers from other ethnicities had 34% higher odds of LBW in Estonia.

The analysis on the effect of mother’s education (Table S6) shows that compared to mothers with high school education, mothers with lower education had higher chances of ABOs and in Ida-Viru County, the odds of having PTB were generally higher by 50% in basic (OR = 2.77, 95% CI: 1.26–5.35) and by 97% in secondary (OR = 2.36, 95% CI: 1.89–2.94) education than in Estonia—OR = 2.27 (95% CI: 1.86–2.75) for basic and OR = 1.39 (95% CI: 1.28–1.51) for secondary education. Ida-Viru ICS had an almost double LBW risk (OR = 3.32, 95% CI: 1.73–5.88 for basic and OR = 2.45, 95% CI: 2.00–3.00 for secondary) than on average in Estonia (OR = 2.48, 95% CI: 2.05–2.99 for basic and OR = 1.59, 95% CI: 1.47–1.73 for secondary).

## 4. Discussion

The current study aimed to investigate perinatal health inequalities in the ICS of Estonia, Ida-Viru oil shale industrial region. The results reported above show a higher prevalence of LBW and PTB in Ida-Viru County than at the national level. Perinatal health inequality is demonstrated through the significantly higher odds ratio in the industrial region of Ida-Viru across most of the studied variables. The results indicate that exposure to PM_2.5_ during the first trimester increased the risks of PTB at the national level, while exposure during the third trimester increased the risks of LBW in the Ida-Viru region. Exposure to BaP during whole pregnancy also affected the LBW in Ida-Viru ICS. Living close to the oil shale industries significantly affected the birth outcomes.

Further analysis of the sociodemographic factors revealed that LBW is associated with ethnicity—Russian and other non-Estonian mothers have higher odds of ABOs. A striking contrast for increased risks of ABOs in Ida-Viru County than at the national level is observed with the mother’s education. Mothers with basic and secondary education are more affected by ABOs; however, the chances are even higher in Ida-Viru.

The identified association between PM_2.5_ and PTB is consistent with other studies [21,22,27,29]. The effects are seen only during the first trimester for PTB in Estonia and the third trimester for LBW in Ida-Viru. Similarly, the systematic review by Sapkota et al. [72] found that PM_2.5_ exposure during the first trimester increased the PTB risks, while the LBW results were inconclusive. On the other hand, the comprehensive review by Klepac et al. [73] reported heterogeneity with both first and third-trimester exposure and PTB, whereas the review and meta-analysis by Ji et al. [74] showed a higher publication bias on the association between LBW and third-trimester PM_2.5_ exposure. Thus, studies seem to have a heterogenous association with ABOs on the trimester-specific PM_2.5_ exposure.

The current study showed a positive but statistically non-significant association between prenatal PM_10_ exposure and ABOs, which is consistent with the existing evidence on non-significant association [72,74]. Despite the inconsistent results shown by earlier reviews [72,74,75], according to the recent review [26], a number of studies conducted in the ICS have shown a significant association between PM_10_ and PTB [18,27,29,35] and LBW [27,29,32,33,53]. One of the reasons for the currently established non-significant association with PM_10_ could be that the average annual concentration is on the borderline of the recommended air quality guideline by WHO [71]. Similarly, the low levels of NO_2_ might explain the no effect of NO_2_ (in the unadjusted model, even protective effect) that is in line with inconsistent results on NO_2,_ including the protective effect identified in a systematic review by Jacobs et al. [75].

The effect of BaP is seen only in LBW in Ida-Viru ICS; thus, the observed relations with air pollution may be coming from other unmeasured pollutants or a mixture of multiple pollutants as well, for instance, PAHs has shown even a strong effect on ABOs as suggested by systematized evidence [26].

Our result on the association between ABOs and proximity to ICS is consistent with previous research [28,38,39,40,41,42]. Living within 2.5 km of a shale gas well is shown to be associated with LBW, while the additional facility increased the association of LBW and PTB by 7% and 3%, respectively [38]. Thus, the effect might be intense among those whose distance is closer to multiple oil shale industrial areas in Ida-Viru. The odds are increasing with a decrease in the distance, indicating higher risks near ICS. The ≤3 km buffer zone consists mainly of residents from Kiviõli town and some of the residents from Kohtla-Järve. Apart from the closer buffer zones to industry, Kiviõli have multiple problems, such as poor environmental quality and high social inequality [76].

The current analysis of the mother’s sociodemographic characteristics showed a higher chance of ABO risk. A possible explanation is that the low SES mothers are more likely to live closer to the ICS, thus exposed to higher amounts of air pollutants [77]. LBW is affected by ethnicity showing similar effects at both national and Ida-Viru levels across Russian ethnicity, but not with the PTB, contrary to previous findings on race/ethnicity and ABOs [50,51]. Differences in ethnicity measures are reported to vary by birth outcomes; PTB effects are more frequently reported than LBW [14]. The pathways for association with ethnicity could be the existing socioeconomic inequalities and a weak sense of belonging among the Russian minorities [66].

A review article in the United States identified two pathways for ethnicity and health [78], which could also shed light on the Estonian context. One of them is institutional disparity influencing access to quality resources. Another is the cultural difference at both individual and societal levels that further promotes the institutional disparity hampering ethnic minority’s economic status and psychological health response [78]. Similarly, another recent U.S.-based study showed that the low SES African–American pregnant mothers are shown to have higher inflammatory profiles, possibly coming from social stressors, which partially mediated the PTB and LBW risk [79]. Hence, the built social and physical environment may modify the psychological health response mediating the ABOs risks, which has currently grasped the attention in social epigenetics [3]. Thus, it may be possible that the psychological health response may be greater among mothers in ICS, given the higher risk perception and the low SES among people in Ida-Viru, including a weak sense of belonging among Russians [61,65,66].

The strong positive association with lower education could be attributed to socioeconomic health pathways [2]. Lower SES mothers are more likely to use substances and cigarettes, have bacterial vaginosis, work strenuously, have psychological problems, whereas they are less likely to take sufficient micronutrients, have adequate prenatal care, and have sufficient leisure time exercise [77,80]. Similarly, pregnant mothers with lower education may have poor working conditions, which is shown to increase PTB risks [81], as lower SES mothers often have poor working conditions [80,82].

Furthermore, ABOs can also be explained via intergenerational transmission of inequalities, where adverse childhood health leads to increased risks of chronic diseases and lower SES, thus continuing the cycle of adverse child health in offspring [83]. For instance, low SES teens are more likely to have poor educational performance in school but more likely to smoke and continue smoking during pregnancy, affecting the birth outcomes of offspring [84]. Thus, to understand the complexity of health inequality, the life course epidemiological approach [85] has been a promising way to investigate the mother’s life course and their living environment affecting health outcomes. The reasons discussed above imply that the cause of ABOs is complex, consisting of multiple pathways instead of a single cause. Our findings help strengthen the environmental and structural determinants of health [1,2], showing the health inequalities in and within the country.

### 4.1. Limitations and Strengths

One of the major limitations of our study is that we did not include the important confounding variables such as smoking due to the unreliable self–reported data which often underestimates the smoking prevalence [86]. Nevertheless, women in Ida-Viru County smoke more frequently compared to other counties in Estonia [87]. Similarly, unavailability of data on alcohol consumption and nutrition was another issue, which has been reported in other birth records-based studies as well [73]. However, the maternal socioeconomic status can provide a glance at health behavior from the perspectives of socioeconomic health pathways. The findings on PTB may be influenced by the biases related to the ultrasound measurements; however, ultrasound is the best available method of estimating gestational age [88]. Due to the unavailability of the exact address, around 6% of records were excluded from the spatial analysis, and the results of Ida-Viru County might partly be affected by low statistical power.

Additionally, the observed result on air pollutants could be over- or underestimated, although we used combined data from monitoring stations and dispersion models. On the one hand, it may not well-capture personal exposure [89,90]. On the other hand, we did not account for indoor and/or occupational exposure to air pollution due to the lack of information, which has also been a common problem in earlier studies [73].

Nevertheless, this study has several strengths. First, it contains all the birth records of Estonia in the last 15 years (from 2004–2018), extracted from the Estonian birth registry. Thus, the selection bias and uncertainty in estimates are largely minimized, and external validity for the whole region is maximized. We have addressed the potential limitation of spatial measurement and modeling by calculating the buffer zones in the proximity to industrial areas and measuring the association with ABOs, which ultimately compliments the exposure to air pollutants results. Sociodemographic variables are important to identify health inequalities across different populations [79,91]. Thus, to provide the comprehensive picture of the ICS, we made the maternal sociodemographic markers our interest in the exposure variable and did not only treat these as covariates. The results are consistent with the conceptual framework of social determinants of health [2], and the study has great potential for evidence-based policymaking.

### 4.2. Future Research Perspectives

Future studies could investigate the combined effect of different environmental and social markers as the combined effects are often higher [55]. Exposome research may allow for assessing the complex interchange of biological, epigenetic, sociodemographic, and environmental factors contributing to adverse health [92]. Future studies can also explore long-term health consequences as a follow-up of the ABOs among newborns in ICS. For this, the life course epidemiological approach [85] can help explore the socioeconomic disparities in early and adult life stages and how a change in the environment (both environmental and social) affects the chances of health consequences.

Given the long-term consequences of ABOs and health inequality, setting up the health monitoring system in the ICS of Ida-Viru is crucial for evidence-based policymaking. Policymakers may strive for equitable policy actions with special emphasis on those differentially exposed to more health-harming characteristics and address the disproportionate distribution of ABOs. PM_2.5_ associations with ABOs demonstrate the need to reduce air pollution levels. Furthermore, association with the proximity to the oil shale industry demands rethinking the role of industries because the evidence showing shutting down the industries leading to positive health effects is not new. Policy actions should be comprehensive and multisectoral with a prime focus on addressing the social stressors to break the chain of health inequality. The observed association of ABOs with education and ethnicity requires action to investigate and address the possible institutional and socio–cultural disparities.

## 5. Conclusions

We found nationwide significant perinatal health inequalities across chemical, physical, and sociodemographic characteristics. However, the inequalities are even more prevalent in Ida-Viru ICS across air quality, residential proximity to oil shale industries, and maternal education. Though in terms of ethnicity, Russian mothers have equally higher chances of ABOs risks regardless of where they live in Estonia. Considering these health disparities in birth outcomes across environmental, residential, and sociodemographic markers, future public health impact across life course might be higher, potentially widening the social disparities. Thus, in addition to reducing the air pollutant level, policy actions on reducing social disparities are vital to address the unjustly higher perinatal health inequalities in the country and the industrial region of Ida-Viru.

## Figures and Tables

**Table 1:** Prevalence of adverse birth outcomes and distribution across the sociodemographic and spatial metrics in Ida-Viru County and Estonia among singleton babies born in 2004–2018.

	Ida-Viru County			Estonia		
	All Births	LBW ^1^	PTB ^2^	All Births	LBW	PTB
**Infant gender, % (*n*)**						
Male	51.3 (9552)	4.4 (421)	4.4 (423)	51.4 (107,013)	3.0 (3217)	3.5 (3724)
Female	48.7 (9074)	5.2 (473)	4.1 (372)	48.6 (101,300)	3.6 (3628)	3.0 (3053)
**Mother’s ethnicity, % (*n*)**						
Estonian	16 (2988)	4.1 (122)	4.1 (122)	72.3 (150,514)	3.0 (4512)	3.1 (4711)
Russian	80.1 (14,906)	5.0 (739)	4.3 (644)	25.1 (52,270)	4.0 (2109)	3.6 (1867)
Others	3.9 (723)	4.4 (32)	3.9 (28)	2.6 (5310)	4.1 (217)	3.6 (192)
**Mother’s education, % (*n*)**						
Basic	0.7 (125)	10.4 (13)	7.2 (9)	1.0 (2176)	6.7 (145)	6.0 (131)
Secondary	13.8 (2573)	9.0 (231)	7.3 (188)	13.2 (27,476)	4.8 (1312)	4.2 (1161)
Higher secondary	25.1 (4682)	4.4 (208)	3.8 (179)	24.0 (49,979)	3.3 (1673)	3.4 (1683)
Applied education	42.3 (7870)	4.2 (333)	3.9 (305)	30.6 (63,753)	3.2 (2021)	3.2 (2021)
Higher education	18.1 (3370)	3.2 (108)	3.4 (113)	31.1 (64,840)	2.6 (1688)	2.7 (1774)
**Mother’s age in years, % (*n*)**						
≤20	5.5 (1025)	6.5 (67)	6.0 (61)	3.7 (7632)	5.1 (392)	4.7 (361)
20–24	24.6 (4589)	4.5 (206)	3.4 (154)	17.8 (37,126)	3.3 (1232)	3 (1115)
25–29	30.5 (5681)	4.4 (248)	3.9 (221)	31.8 (66,307)	2.9 (1908)	2.8 (1889)
30–34	23.4 (4362)	4.7 (203)	4.1 (181)	27.7 (57,760)	3.0 (1721)	3.1 (1795)
35–39	12.7 (2368)	5.3 (125)	5.5 (130)	14.9 (30,990)	3.7 (1162)	3.8 (1190)
>40	3.2 (601)	7.5 (45)	8.0 (48)	4.1(8496)	5.1 (430)	5.0 (427)
**Proximity to industrial areas ^3^, % (*n*)**						
≤3 km	10.1 (1791)	5.4 (96)	4.8 (86)	0.99 (1816)	5.3 (97)	4.8 (88)
≤5 km	43.4 (7699)	4.9 (375)	4.2 (321)	4.2 (7724)	4.9 (376)	4.2 (323)
≤10 km	61.9 (10,992)	4.6 (508)	4.0 (444)	6.0 (11,033)	4.6 (509)	4.0 (446)

^1^ Low birth weight; ^2^ Preterm birth; ^3^ Cumulative observations with increasing proximity.

**Table 2:** Associations between adverse birth outcomes and exposure to air pollutants (per 10 µg/m^3^) during the first and third trimesters in Estonia and Ida-Viru County.

	Ida-Viru County		Estonia		
	PM_10_	PM_2.5_	PM_10_	PM_2.5_	NO_2_
	OR (95% CI)				
**Preterm birth (PTB)**					
**Adjusted model ^2^**					
I trimester	1.10 (0.98–1.24)	1.01 (0.73–1.39)	1.07 (1.02–1.13)	**1.09 ^1^** **(1.00–1.20)**	1.02 (0.94–1.10)
III trimester	1.09 (0.97–1.23)	1.26 (0.92–1.72)	1.03 (0.97–1.09)	1.01 (0.92–1.11)	0.91 (0.84–0.99)
**Fully–adjusted model ^3^**					
I trimester	1.12 (0.99–1.26)	0.99 (0.70–1.37)	1.05 (0.99–1.11)	**1.12** **(1.02–1.23**)	1.04 (0.96–1.13)
III trimester	1.12 (0.98–1.26)	1.30 (0.95–1.79)	1.03 (0.97–1.09)	1.03 (0.94–1.13)	0.90 (0.83–0.98)
**Low birth weight (LBW)**					
**Adjusted model**					
I trimester	1.10 (0.98–1.23)	0.99 (0.73–1.35)	1.05 (0.99–1.11)	1.05 (0.96–1.15)	0.96 (0.88–1.03)
III trimester	1.04 (0.93–1.17)	**1.50** **(1.12–1.99)**	1.05 (0.93–1.17)	1.02 (0.93–1.13)	0.93 (0.85–1.01)
**Fully–adjusted model**					
I trimester	1.12 (0.99–1.25)	0.96 (0.70–1.33)	1.02 (0.97–1.08)	1.06 (0.97–1.17)	0.98 (0.91–1.06)
III trimester	1.06 (0.94–1.19)	**1.56** **(1.16–2.08)**	1.06 (0.94–1.19)	1.03 (0.94–1.14)	0.92 (0.85–1.00)

^1^ Bold values are statistically significant results; ^2^ Adjusted for individual-level sociodemographic variables (mother’s ethnicity, mother’s education, mother’s age); ^3^ Adjusted for individual-level sociodemographic variables from adjusted model and maternal health status (in vitro fertilization, earlier cesarean section, preeclampsia, preterm birth risk during pregnancy, miscarriage risk during pregnancy, gestational diabetes, chronic diabetes, and hypertension).

**Table 3:** Associations between adverse birth outcomes and mother’s residential proximity to oil shale industries in Estonia.

Residential Proximity	≤3 km ^1^	≤5 km ^1^	≤10 km ^1^
		OR (95% CI)	
**Preterm birth (PTB)**			
Adjusted model ^3^	**1.54 ^2^** **(1.23–1.90)**	**1.38** **(1.22–1.56)**	**1.35** **(1.22–1.50)**
Fully–adjusted model ^4^	**1.58** **(1.26–1.98)**	1.07 (0.94–1.22)	1.01 (0.89–1.14)
**Low birth weight (LBW)**			
Adjusted model	**1.55** **(1.26–1.91)**	**1.48** **(1.32–1.66)**	**1.42** **(1.29–1.57)**
Fully–adjusted model	**1.51** **(1.21–1.88)**	**1.18** **(1.04–1.33)**	1.09 (0.97–1.23)

^1^ Reference distance is >10 km; ^2^ Bold values are statistically significant results; ^3^ Adjusted for individual-level sociodemographic variables (mother’s ethnicity, mother’s education, mother’s age); ^4^ Adjusted for individual-level sociodemographic variables from adjusted model and maternal health status (in vitro fertilization, earlier cesarean section, preeclampsia, preterm birth risk during pregnancy, miscarriage risk during pregnancy, gestational diabetes, chronic diabetes, and hypertension), and neighborhood socioeconomic status (income coefficient).

**Table 4:** Associations between adverse birth outcomes and mother’s ethnicity.

	Ida-Viru County		Estonia	
Mother’s Ethnicity	Russians ^1^	Other Non-Estonians ^1^	Russians	Other Non-Estonians
OR (95% CI)				
**Preterm birth (PTB)**				
Adjusted model ^3^	1.09 (0.89–1.33)	0.92 (0.60–1.39)	**1.15 ^2^** **(1.09–1.22)**	1.14 (0.99–1.33)
Fully–Adjusted model ^4^	1.08 (0.88–1.33)	0.93 (0.60–1.41)	1.05 (0.99–1.11)	1.10 (0.94–1.28)
**Low birth weight (LBW)**				
Adjusted model	**1.26** **(1.04–1.54)**	1.09 (0.72–1.61)	**1.36** **(1.29–1.43)**	**1.37** **(1.19–1.57)**
Fully–Adjusted model	**1.29** **(1.05–1.57)**	1.11 (0.74–1.67)	**1.29** **(1.22–1.36)**	**1.34** **(1.15–1.55)**

^1^ Reference population: Mothers of Estonian ethnicity; ^2^ Bold values are statistically significant results; ^3^ Adjusted for the individual–level sociodemographic variables (mother’s education, mother’s age); ^4^ Adjusted for individual-level sociodemographic variables from adjusted model and maternal health status (in vitro fertilization, earlier cesarean section, preeclampsia, preterm birth risk during pregnancy, miscarriage risk during pregnancy, gestational diabetes, chronic diabetes, and hypertension), and neighborhood socioeconomic status (income coefficient).

## Data Availability

Data are available on request from the Estonian Birth Registry.

## Supplementary Materials

The following supporting information can be downloaded at: https://www.mdpi.com/article/10.3390/ijerph191811559/s1.
