# Supplementary material for: Perinatal Health Inequalities in the Industrial Region of Estonia: A Birth Registry-Based Study
Source: Int J Environ Res Public Health. 2022 Sep 14;19(18):11559. doi: 10.3390/ijerph191811559 (PMC9516979; doi:10.3390/ijerph191811559)
Supplement: Supplementary file 1 [file ijerph-19-11559-s001.zip › ijerph-1863987-supplementary.pdf]

Supplementary material

Perinatal health inequalities in the industrial region of Estonia: a birth registry-based study, by Usha Dahal, Triin Veber, Daniel Oudin Åström, Tanel Tamm, Leena Albreht, Erik Teinemaa, Kati Orru and Hans Orru

List of Figures

Figure S1. Annual average concentrations (μg/m^3) of particulate matter (PM_{10}), fine particles ($PM_{2.5}$), and nitrogen dioxide (NO_2) in air quality monitoring stations in Estonia.

Figure S2. Modeled annual average concentrations of benzo(a)pyrene in Ida-Viru County.

List of Tables

Table S1. Average exposure to air pollutants (μg/m^3) during the first and third trimesters.

Table S2. Crude model on associations between adverse birth outcomes and exposure to air pollutants (per 10 μg/m^3) during the first and third trimesters in Estonia and Ida-Viru County.

Table S3. Associations between adverse birth outcomes and exposure to benzo(a)pyrene (per 10 μg/m^3) during whole pregnancy in Ida-Viru County.

Table S4. Crude model on associations between adverse birth outcomes and mother's residential proximity to oil shale industries in Estonia.

Table S5. Crude model on associations between adverse birth outcomes and mother's ethnicity.

Table S6. Associations between adverse birth outcomes and mother's educational level.

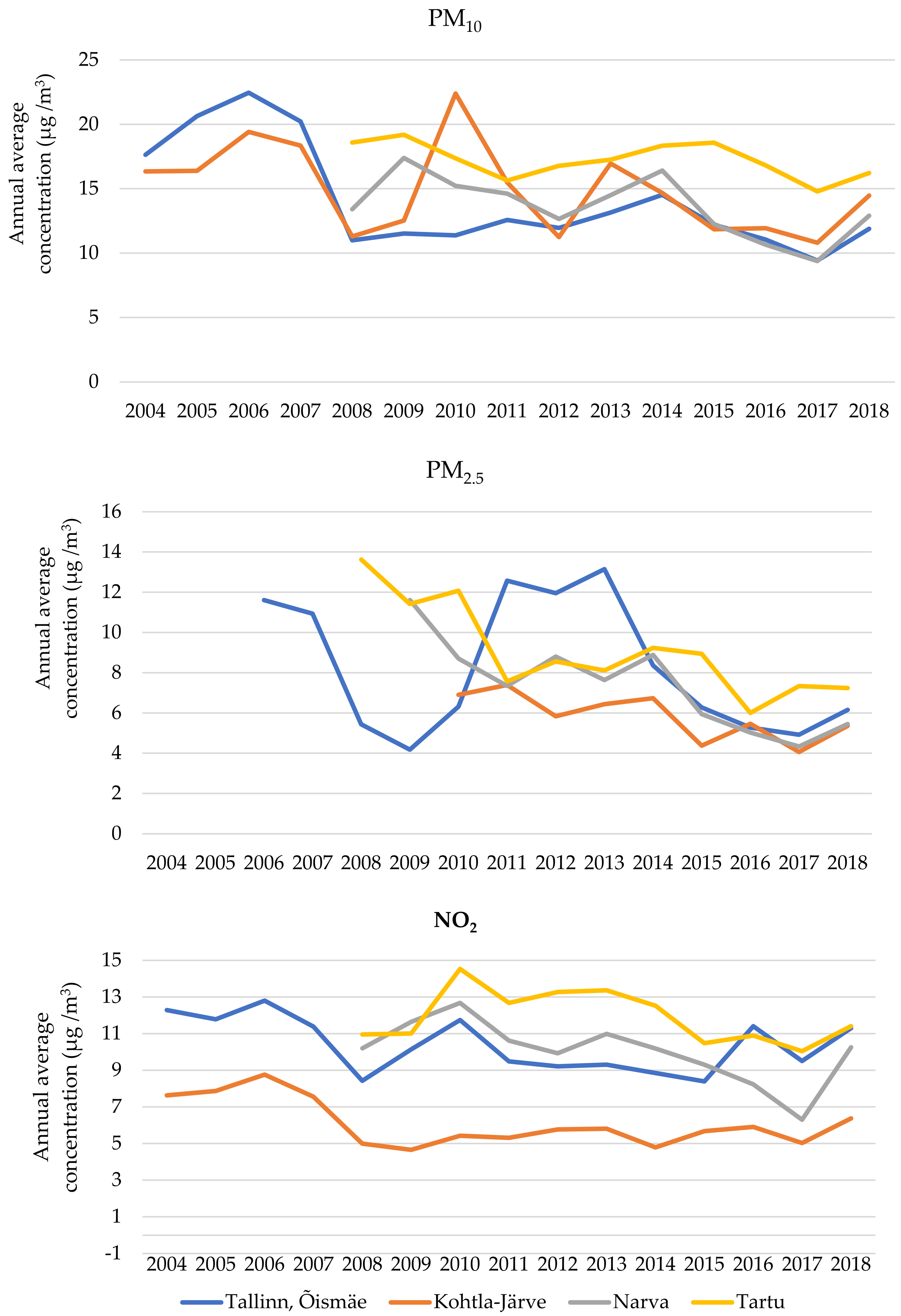

Figure S1. Annual average concentrations (μg/m³) of particulate matter (PM_{10}), fine particles ($PM_{2.5}$), and nitrogen dioxide (NO_2) in air quality monitoring stations in Estonia.

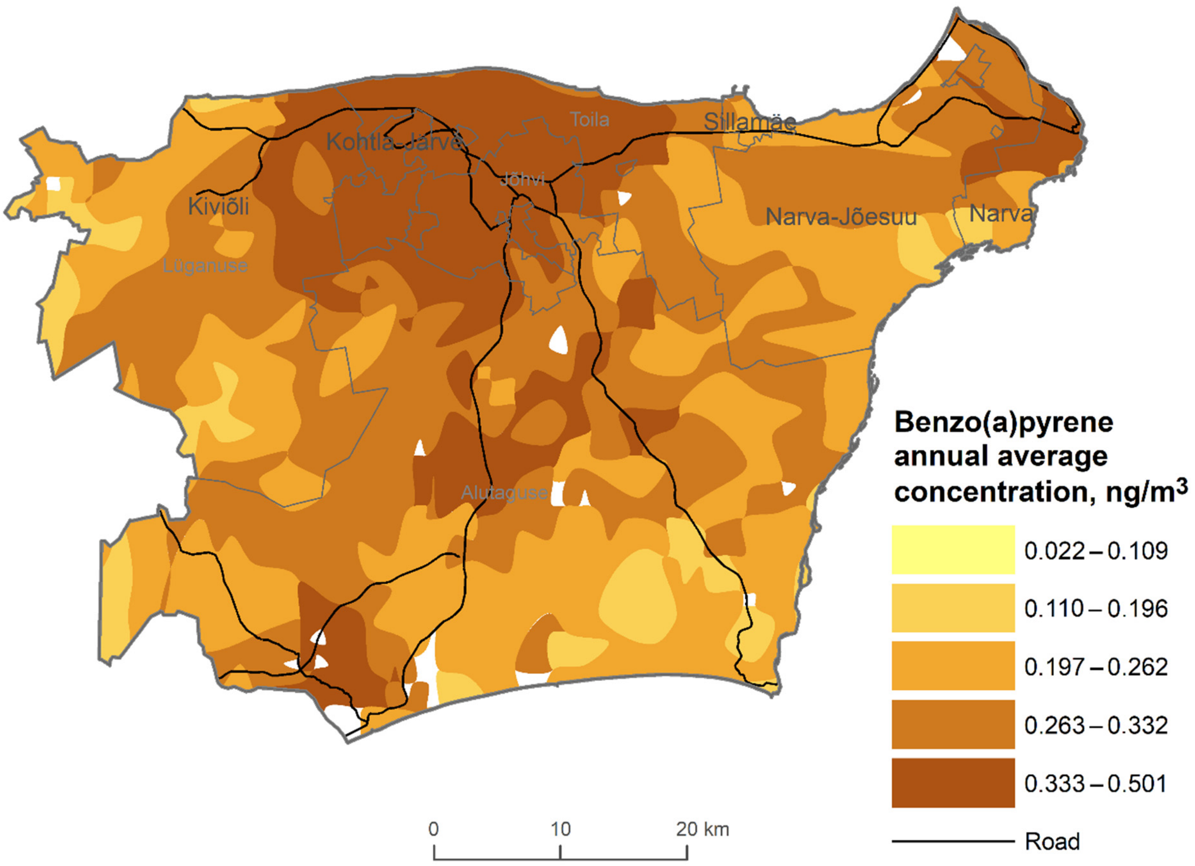

Figure S2. Modeled annual average concentrations of benzo(a)pyrene in Ida-Viru County.

Table S1. Average exposure to air pollutants (μg/m³) during the first and third trimesters.

Pollutant	Ida-Viru County						Estonia					
	PM_{10}		$PM_{2.5}$		NO_2		PM_{10}		$PM_{2.5}$		NO_2	
**Trimester**	**I**	**III**	**I**	**III**	**I**	**III**	**I**	**III**	**I**	**III**	**I**	**III**
**Term birth with normal weight**	16.0	16.1	8.7	8.2	7.7	7.6	12.4	12.4	6.9	6.8	8.9	8.8
**Low birth weight (LBW)**	16.5	16.5	8.7	8.6	7.6	8.1	12.4	12.3	6.9	6.8	8.8	8.7
**Preterm birth (PTB)**	16.4	16.7	8.7	8.4	8.0	7.8	12.5	12.3	6.9	6.7	8.9	8.7

Table S2. Crude model on associations between adverse birth outcomes and exposure to air pollutants (per 10 μg/m^3) during the first and third trimesters in Estonia and Ida-Viru County.

Air Pollutants	Ida-Viru County		Estonia		
	PM_{10}	$PM_{2.5}$	PM_{10}	$PM_{2.5}$	NO_2
	OR (95% CI)				
**Preterm birth (PTB)**					
**Crude model**					
I trimester	1.08 (0.96–1.21)	0.94 (0.68–1.29)	1.05 (0.99–1.11)	1.06 (0.97–1.16)	0.98 (0.90–1.05)
III trimester	1.07 (0.95–1.21)	1.20 (0.88–1.63)	1.01 (0.95–1.07)	0.99 (0.90–1.08)	0.89 (0.82–0.96)
**Low birth weight (LBW)**					
**Crude model**					
I trimester	1.09 (0.97–1.22)	0.94 (0.68–1.27)	1.04 (0.98–1.10)	1.04 (0.94–1.13)	**0.91**[1] **(0.84–0.99)**
III trimester	1.04 (0.92–1.16)	**1.45 (1.08–1.92)**	0.99 (0.93–1.05)	1.01 (0.92–1.11)	**0.90 (0.83–0.97)**

[1]Bold values are statistically significant results

Table S3. Associations between adverse birth outcomes and exposure to benzo(a)pyrene (per 10 ng/m^3) during whole pregnancy in Ida-Viru County.

**Benzo(a)pyrene**	**Ida-Viru**
	**OR (95% CI)**
**Preterm birth (PTB)**	
Crude model	1.18 (0.45-3.21)
Adjusted model[2]	1.31 (0.49-3.57)
Fully-adjusted model[3]	2.04 (0.73-5.75)
**Low birth weight (LBW)**	
Crude model	2.57 (0.99-6.72)
Adjusted model	2.59 (0.99-6.83)
Fully-adjusted model	**4.06 (1.50-11.1)**[1]

[1]Bold values are statistically significant results; [2]Adjusted for individual-level sociodemographic variables (mother's ethnicity, mother's education, mother's age); [3]Adjusted for individual-level sociodemographic variables from adjusted model and maternal health status (in–vitro fertilization, earlier cesarean section, preeclampsia, preterm birth risk during pregnancy, miscarriage risk during pregnancy, gestational diabetes, chronic diabetes, and hypertension).

Table S4. Crude model on associations between adverse birth outcomes and mother's residential proximity to oil shale industries in Estonia.

**Residential proximity**	**≤ 3 km**	**≤ 5 km**	**≤ 10 km**
		**OR (95% CI)**	
**Preterm birth (PTB)**			
Crude model	**1.63**[1] **(1.31–2.02)**	**1.41 (1.26–1.58)**	**1.37 (1.24–1.51)**
**Low birth weight (LBW)**			
Crude model	**1.81 (1.48–2.23)**	**1.68 (1.51–1.87)**	**1.60 (1.45–1.75)**

[1]Bold values are statistically significant results

Table S5. Crude model on associations between adverse birth outcomes and mother's ethnicity.

Mother`s Ethnicity	Ida-Viru County		Estonia	
	**Russians[1]**	**Other non-Estonians[1]**	**Russians[1]**	**Other non-Estonians[1]**
	**OR (95% CI)**			
**Preterm birth (PTB)**				
Crude model	1.06 (0.87-1.30)	0.95 (0.61–1.42)	**1.15[2] (1.09–1.21)**	**1.16 (1.00–1.35)**
**Low birth weight (LBW)**				
Crude model	**1.23 (1.01–1.49)**	1.09 (0.72–1.60)	**1.36 (1.29–1.43)**	**1.38 (1.20–1.58)**

[1]Reference population: Mothers of Estonian ethnicity; [2]Bold values are statistically significant results.

Table S6. Associations between adverse birth outcomes and mother's educational level.

Mother's Education	Ida-Viru County				Estonia			
	Basic[1]	Secondary[1]	Applied[1]	Higher[1]	Basic	Secondary	Applied	Higher
	OR (95% CI)							
**Preterm Birth (PTB)**								
Crude model	**1.95**[2] **(0.91–3.70)**	**1.98 (1.61–2.45)**	1.01 (0.84–1.23)	**0.87 (0.69–1.11)**	**1.84 (1.52–2.20)**	**1.27 (1.17–1.37)**	0.94 (0.88–1.00)	**0.81 (0.75–0.86)**
Adjusted model[3]	**2.53 (1.17–4.82)**	**2.35 (1.90–2.92)**	0.91 (0.76–1.11)	**0.73 (0.57–0.93)**	**2.23 (1.85–2.68)**	**1.43 (1.32–1.55)**	**0.90 (0.84–0.96)**	**0.74 (0.69–0.80)**
Fully–adjusted model[4]	**2.77 (1.26–5.35)**	**2.36 (1.89–2.94)**	0.91 (0.75–1.11)	**0.70 (0.55–0.90)**	**2.27 (1.86–2.75)**	**1.39 (1.28–1.51)**	**0.89 (0.83–0.96)**	**0.78 (0.73–0.84)**
**Low Birth Weight (LBW)**								
Crude model	**2.50 (1.32–4.35)**	**2.12 (1.75–2.58)**	0.95 (0.80–1.14)	0.95 (0.80–1.14)	**2.06 (1.73–2.45)**	**1.45 (1.34–1.56)**	0.95 (0.89–1.01)	**0.77 (0.72–0.82)**
Adjusted model	**3.02 (1.59–5.29)**	**2.42 (1.98–2.95)**	0.88 (0.74–1.05)	**0.62 (0.49–0.79)**	**2.44 (2.05–2.92)**	**1.64 (1.52–1.77)**	**0.92 (0.86–0.98)**	**0.73 (0.68–0.78)**
Fully–adjusted model	**3.32 (1.73–5.88)**	**2.45 (2.00–3.00)**	0.88 (0.74–1.06)	**0.60 (0.47–0.76)**	**2.48 (2.05–2.99)**	**1.59 (1.47–1.73)**	**0.91 (0.85–0.97)**	**0.77 (0.72–0.83)**

[1]Reference population: Mothers with high school level education; [2]Bold values are statistically significant results; [3]Adjusted for individual-level sociodemographic variables (mother's ethnicity, mother's age); [4]Adjusted for individual-level sociodemographic variables from adjusted model and maternal complications (in–vitro fertilization, earlier cesarean section, preeclampsia, preterm birth risk during pregnancy, miscarriage risk during pregnancy, gestational diabetes, chronic diabetes, and hypertension), and neighborhood socioeconomic status (income coefficient).
